# Validation of the Transformer-Based Monocular System (Capture4D): A Real-Time Kinematic Analysis in Coaching/Teaching Tennis

**DOI:** 10.3390/s26051411

**Published:** 2026-02-24

**Authors:** Yue Zhao, Shuo Wang, Zan Gao, Haijun Wu, Yixiong Cui, Yuanlong Liu

**Affiliations:** 1 Sports Coaching College, Beijing Sport University, Beijing 100084, China; 2 Beijing PinXi Technology Co., Ltd., Beijing 100089, China; shuo-wan19@mails.tsinghua.edu.cn; 3 Department of Kinesiology, Recreation, and Sport Studies, The University Tennessee, Knoxville, TN 37996, USA; zan@utk.edu; 4 China Institute of Sport and Health Science, Beijing Sport University, Beijing 100084, China; haijunwu@bsu.edu.cn; 5 School of Sports Engineering (China Sports Big Data Center), Beijing Sport University, Beijing 100084, China; cuiyixiong@bsu.edu.cn; 6 Department of Human Performance and Health Education, Western Michigan University, Kalamazoo, MI 49008, USA

**Keywords:** computer vision, human motion capture, tennis, monocular camera, transformer

## Abstract

Human motion capture is crucial for various fields, but traditional optical systems (OMC) are costly and restrictive. Monocular video-based methods offer accessibility, yet face accuracy challenges, especially in dynamic sports like tennis. This study validates Capture4D, a novel Transformer-based monocular system, for capturing a wide range of tennis strokes. We developed a universal biomechanical analysis framework (K0-K5) applicable to twelve fundamental stroke types. To demonstrate the system’s capabilities, this paper focused on a detailed validation using the tennis serve as a representative example. We conducted experiments with 9 high-level tennis players, and motion data were simultaneously captured using Capture4D (single RGB camera) and OMC Qualisys (gold standard). Accuracy was evaluated by comparing 3D joint coordinates and joint angles using Normalized Mean Per Joint Position Error (NMPJPE), RMSE, and MAE. The results demonstrated that Capture4D effectively captured the tennis player’s motion, with average NMPJPE for tennis serves ranging from 69.5 mm to 88.3 mm, within the acceptable range (70–130 mm) for coaching purposes. Compared to OMC, Capture4D demonstrated comparable joint angle trajectories, with advantages in operational convenience, cost-effectiveness, and wider applicability. It offered an approximately 50% reduction in setup time and 80% cost savings. Capture4D presents a valid and practical monocular motion capture solution for coaching tennis and other broader applications in sports. While slightly less precise than OMC, its accuracy is acceptable for many use cases in coaching and teaching. It offers significant advantages in convenience and cost, paving the way for accessible motion analysis in diverse environments like outdoor settings and multi-person scenarios, in which OMC is not possible to be used. This technology holds promise for democratizing motion capture in sports training and coaching/teaching.

## 1. Introduction

Quantitative analysis of human movement is a cornerstone of modern sports science, providing critical insights for performance enhancement and injury prevention [1]. In net sports such as tennis, the precise measurement of three-dimensional (3D) kinematics—including joint angles, segment velocities, and spatial positioning—is essential for coaches and scientists to understand and optimize complex motor skills [2]. For decades, Optical Motion Capture (OMC) systems have been accepted as a “gold standard” for this purpose, offering unparalleled accuracy in controlled laboratory environments [3].

However, the high cost, operational complexity, and environmental constraints of OMC systems create a significant “lab-to-field” gap. The data captured in a sterile lab setting often fail to represent the variable and dynamic conditions of on-court performance, limiting the ecological validity and practical applicability of the findings for coaches and athletes. Factors such as marker slippage during high-intensity movements, the psychological effect of being in a lab environment, and the inability to capture data during actual competition scenarios are well-known limitations. This gap highlights a pressing need for accessible, portable, and cost-effective motion analysis tools that can be deployed “in the wild” without encumbering the athlete or disrupting the natural training/competition environment.

Marker-less motion capture systems [4,5,6,7,8], particularly those using a single, readily available video camera (monocular systems) [9,10,11,12,13,14,15,16], present a promising solution to bridge this gap. The advent of deep learning, particularly Convolutional Neural Networks (CNNs), revolutionized the field by enabling robust 2D key point detection from images [17], with systems such as OpenPose becoming widely adopted [18] in coaching and training environments.

More recently, the Transformer architecture, which has shown remarkable success by modeling global context through self-attention mechanisms [19] has been adapted for computer vision tasks [20]. This study focused on validating Capture4D, a novel monocular motion capture system that leverages a powerful Transformer-based architecture, similar in principle to pioneering works like 4DHumans [21]. Unlike traditional CNNs, this architecture is specifically designed to better model the holistic relationship among all body segments simultaneously, offering the potential for more robust 3D pose estimation [22], even during complex and rapid athletic movements in competitions.

The purpose of this research was to conduct a rigorous validation of the Capture4D system for 3D kinematic analysis across a comprehensive tennis strokes. This paper presents the detailed validation for the serve, which represents a particularly challenging use-case due to its high velocity and complexity. To our best knowledge, this study could be the first to systematically validate a Transformer-based monocular motion capture system for on-court tennis stroke analysis. It provides a practical and accessible solution to bridge the lab-to-field gap in sport analysis for coaches, teachers, and athletes. Our objective was to determine whether Capture4D could provide an acceptable level of accuracy in coaching/teaching and biomechanical research. It could offer a practical and accessible alternative to fill the gap between traditional laboratory-based and on court environment evaluation. We hypothesize that Capture4D would achieve sufficient accuracy in joint kinematics to enable in-field coaching/teaching applications, with temporal agreement comparable to gold-standard OMC systems.

## 2. Methods

### 2.1. The Capture4D System: Core Architecture and Training

Capture4D is a markerless monocular human motion capture system based on a Transformer deep learning architecture. Its primary objective is to reconstruct temporally coherent 3D human pose and shape from 2D video recorded by a single, standard camera.

#### 2.1.1. Model Architecture

The technical core of the system is a fully “Transformerized” neural network, which includes a Vision Transformer (ViT) encoder and a cross-attention-based Transformer decoder:

1. ViT Encoder: The system employs a powerful ViT-H/16 model as its image encoder. For each input video frame, the encoder first decomposes it into a series of image patches and then extracts image features representing global contextual information through its multi-layer self-attention mechanism. This enables the model to effectively capture long-range spatial dependencies among different body parts, which is crucial for handling complex poses and severe occlusions.
2. Transformer Decoder: The image features extracted by the encoder are fed into a 6-layer decoder. The decoder interacts with the image features via a cross-attention mechanism, using a learnable query that represents the human model. It ultimately regresses a set of SMPL (Skinned Multi-Person Linear Model) parameters [23], including pose parameters (θ) defining body posture, shape parameters (β) defining height and build, and camera parameters (π) relative to the person.

#### 2.1.2. Training Data

To ensure robustness and generalization in real-world scenarios, Capture4D’s training process utilizes a large-scale, diverse hybrid dataset strategy. This includes: (1) datasets with precise 3D ground truth, such as Human3.6M [24] and MPI-INF-3DHP [25], for learning accurate 3D human structure; and (2) large-scale “in-the-wild” image datasets with only 2D annotations, such as COCO [26], AI Challenger [27], and InstaVariety [28], to enhance the model’s recognition capabilities under complex backgrounds, lighting, and clothing conditions [29].

### 2.2. Experimental Validation: Participants and Protocol

#### 2.2.1. Participants

Nine elite-level tennis players (Level 1 athletes and above) were recruited for this study. All participants were right-handed, with an average of 12 years of professional training experience (training duration: 12 ± 3 years, where “±3 years” represents the standard deviation), indicating a solid and systematic professional training background. Given the scarcity of the high-level professional tennis player population and the high demand for data quality, similar to this type of study in the literature, the sample size of 9 is practically feasible and scientifically reasonable. While the sample size is relatively small, it is consistent with standards in high-performance sports biomechanics research where elite populations are scarce and possess highly homogeneous, stable movement patterns. This ensures the reliability of the validation within the target user group. Prior to data collection, all participants were fully informed of the experimental procedures and objectives and provided written informed consent. This study protocol was approved on 9 October 2025 by the Beijing Sport University Institutional Review Board (BSU IRB) with CIRBID 2025426H.

#### 2.2.2. Apparatus and Protocol

Since the OMC could not be set up outdoors, to compare the two systems (Table 1), this study was conducted indoors in an open room of approximately 10 m by 10 m with a flat, non-slip floor. The experimental environment was set up with sufficient, uniform lighting and a simple background.

The following equipment was used:

1. Monocular Camera: Used to record the participants’ motion videos as input for the Capture4D system.
2. Optical Motion Capture (OMC) System: A Qualisys system was used as the “gold standard” for data collection to validate the accuracy of the Capture4D system. The OMC system includes high-speed cameras and is equipped with professional capture and data processing software.
3. Reflective Markers: Used by the OMC system to capture human motion information. Markers were placed on key anatomical landmarks, such as the head, shoulders, elbows, wrists, hips, knees, and ankles.
4. Data Acquisition Computer: Used to store and process experimental data, configured to meet the operational requirements of both the monocular camera and the OMC system.
5. Sports Equipment: A tennis racket was used to assist participants in completing the specified actions.

During the data collection phase, participants performed tennis serves.

### 2.3. Experimental Validation: Data Processing and Analysis

The data analysis workflow was designed to achieve two core objectives: (1) to quantitatively validate the overall 3D reconstruction accuracy of the Capture4D system; and (2) to evaluate its effectiveness in analyzing key biomechanical events in practical sports science applications.

**Figure 1:**
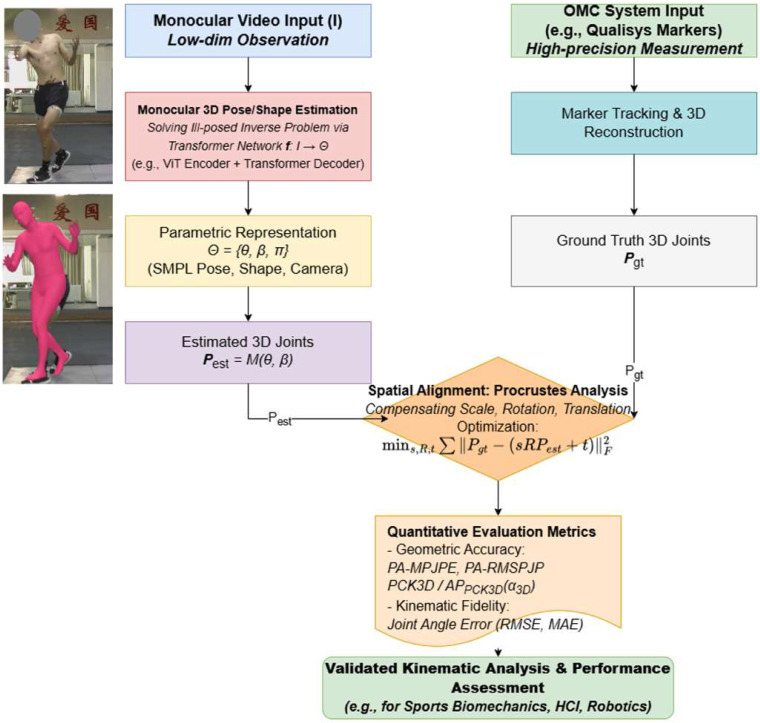
The overall methodological workflow, illustrating the parallel data capture from a single monocular camera (Capture4D) and a gold-standard OMC system, followed by spatial alignment and comparative analysis.

**Figure 2:**
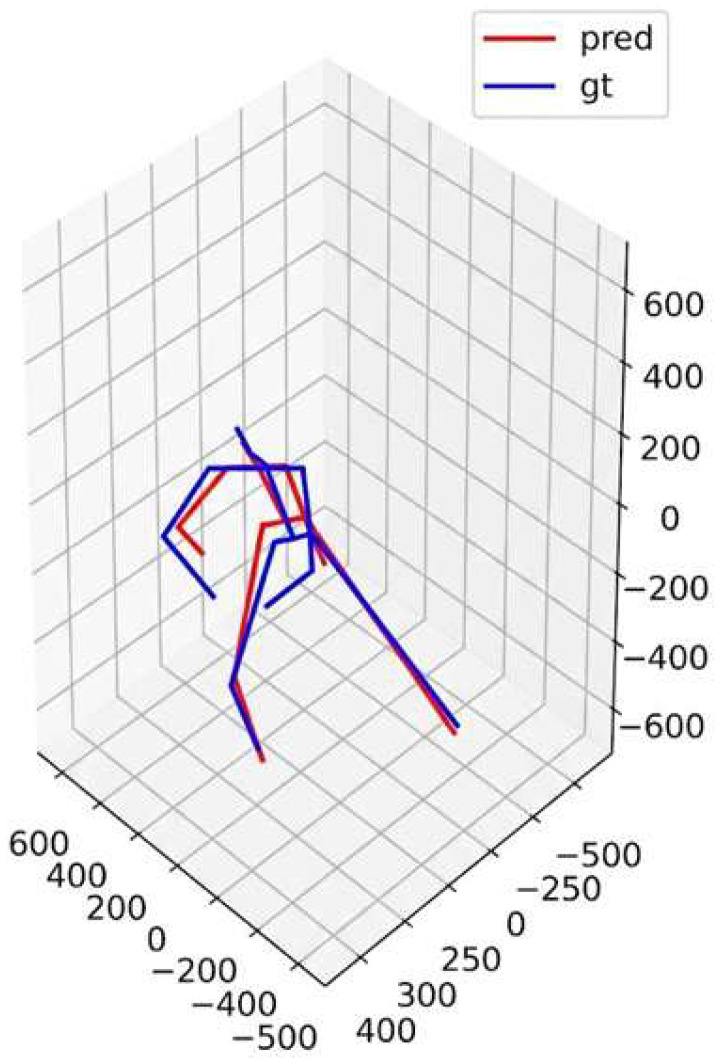
Qualitative evaluation of 3D reconstruction. The red skeleton represents the prediction (pred) from Capture4D, aligned with the blue ground truth (gt) skeleton from the Qualisys OMC system.

1. Data Preprocessing: The raw 3D coordinate data exported from both systems were first temporally synchronized. To reduce the impact of high-frequency noise on kinematic calculations, a Savitzky–Golay smoothing filter was applied to the joint trajectories output by the Capture4D system.
2. Overall Accuracy Validation: To quantify the overall 3D reconstruction accuracy of the Capture4D system, we used the Normalized Mean Per Joint Position Error (NMPJPE) as the primary metric. This method involves aligning the Capture4D-predicted skeleton of each frame with the gold-standard skeleton measured by the Qualisys system through a rigid transformation (rotation, translation, and scaling) via Procrustes analysis. The average Euclidean distance between all corresponding joints is then calculated. This method eliminates systematic errors arising from different coordinate system definitions and scale differences, thereby enabling a fair assessment of the model’s intrinsic accuracy.
3. Applied Biomechanical Analysis: A core component of our validation was the development of a universal analysis framework applicable to all captured movements. We segmented each of the twelve stroke types into five biomechanically significant key phases (K0—Preparation, K1—Backswing start, K2—Peak of Backswing, K3—Contact Point, K4—Follow-through, and K5—Recovery). The identification of these keyframes was based on kinematic criteria derived from the motion data itself, ensuring an objective and reproducible segmentation of the movement. The phases are defined as follows:

The entire data processing and analysis pipeline, summarized in Figure 1 and Figure 2, was implemented using the Python (v3.11.10) programming language, in conjunction with scientific computing and visualization libraries such as Pandas(v2.2.3), NumPy(v1.26.4), joblib(v1.4.2), and Matplotlib(v3.9.2).

## 3. Results

### 3.1. Overall System Accuracy

The overall accuracy of the Capture4D system was first evaluated against the gold-standard OMC system across all captured tennis serve trials. As shown in Table 2, the Normalized Mean Per Joint Position Error (NMPJPE) ranged from 69.5 mm to 88.3 mm. These values fell well within the acceptable error margins (typically 70–130 mm) for applied biomechanical analysis, confirming the system’s foundational validity.

### 3.2. Kinematic Trajectory and Phase Analysis

**Figure 3:**
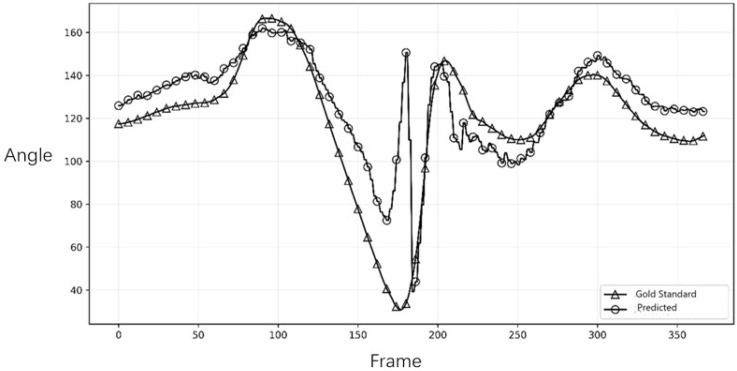
Comparison of the right elbow joint angle trajectory.

**Figure 4:**
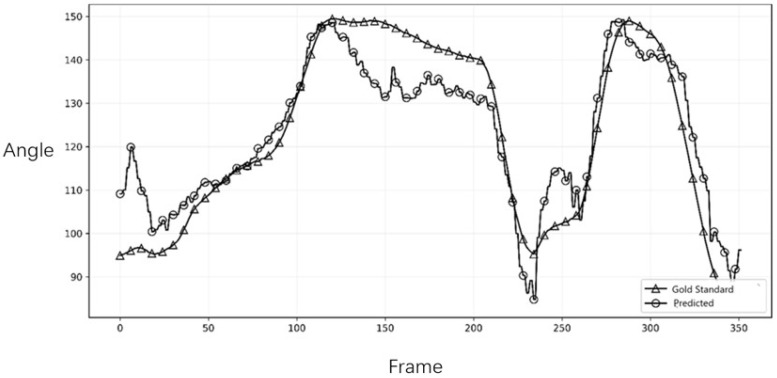
Comparison of the left elbow joint angle trajectory.

To assess the system’s ability to capture the dynamic characteristics of the tennis serve, we compared the joint angle trajectories over time. Figure 3 and Figure 4 illustrate the comparison for the right and left elbow joint from representative trials. The plots demonstrated a high degree of temporal agreement between Capture4D (predicted) and the OMC system (gold standard), with both systems capturing the key kinematic events, peaks, and troughs with minimal phase lag. This indicates that Capture4D can reliably track the temporal dynamics of complex, high-speed movements.

To further evaluate the system’s practical utility for coaching, we developed a comprehensive analysis framework breaking down twelve common tennis strokes (including forehand, backhand, volley, and serve) into biomechanically significant phases. The serve, as an example, was segmented into five key phases, as defined in Table 3, to allow for a targeted analysis relevant to coaching.

### 3.3. Error Distribution Analysis and Biomechanical Interpretation

While the overall trajectory alignment in Figure 3 and Figure 4 demonstrated high temporal consistency, a detailed phase-specific inspection revealed notable deviations in absolute joint angles during specific high-velocity phases. Specifically, the discrepancy observed in the right elbow flexion (Figure 3) peaked during the transition from the “Trophy Pose” to the “Racket Drop” (late K1 to K2). This phenomenon can be attributed to the inherent depth ambiguity of monocular 3D reconstruction. In this phase, the athlete’s arm moves rapidly behind the torso, leading to significant self-occlusion where the limb is partially invisible to the single camera view.

Although the Transformer-based architecture of Capture4D—similar to the mechanisms described in advanced human mesh recovery systems (e.g., HMR 2.0)—leverages global attention to infer occluded body parts from context, the lack of direct depth information inevitably introduces uncertainty. When the forearm aligns parallel to the camera’s optical axis (foreshortening), the system must rely on learned priors rather than geometric triangulation, resulting in the observed variance in the predicted angle magnitude.

However, from a coaching and pedagogical perspective, these absolute positional errors are secondary to kinematic fidelity. Crucially, the system accurately captures the timing of the peak flexion and the subsequent extension velocity. As shown in the plots, the phase shifts (temporal lag) between the predicted and gold-standard curves are negligible. This confirms that Capture4D effectively preserves the kinetic chain sequencing—such as the critical synchronization between hip rotation and arm extension—which is the primary focus in technical diagnosis. Therefore, despite the presence of systematic estimation errors due to occlusion, the system remains a valid tool for identifying technical flaws and monitoring movement patterns in real-world training scenarios

### 3.4. Visual and Positional Fidelity

**Figure 5:**
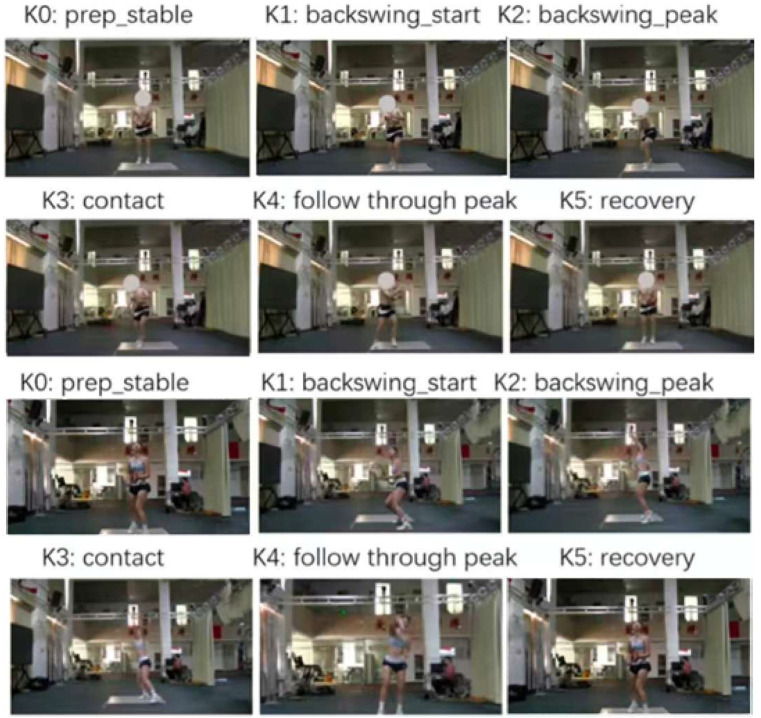
Visual comparison of key pose estimations during the representative tennis skill motions.

Finally, to provide a qualitative assessment of the system’s reconstruction fidelity, Figure 5 presents a visual comparison of the 3D poses at critical keyframes of the serve: K0 (Preparation), K1 (Backswing Start), K2 (Peak Backswing), K3 (Contact), K4 (Follow-through peak), and K5 (Recovery). The visual alignment confirms that Capture4D accurately captures the overall body posture and limb configuration throughout the movement, providing an intuitive and correct representation of the athlete’s technique.

## 4. Discussion

This study shows the acceptable validity of the Capture4D system, a Transformer-based monocular motion capture tool, for biomechanical analysis using tennis serve in the data collection protocol. Our results indicate that while the system’s absolute positional accuracy (NMPJPE) is slightly lower than the gold-standard OMC, its ability to accurately track kinematic trajectories and quantify key kinematic events makes it a powerful and practical tool for sports science applications.

Notably, the system’s portability and single-camera setup extend its utility beyond tennis. It holds potential for other dynamic sports requiring unrestricted movement, such as figure skating or other racket sports, where traditional OMC systems are impractical. By validating Capture4D in tennis, this study demonstrates a versatile solution for calculating joint kinematics in diverse training environments.

### 4.1. Implications for Sports Science and Coaching/Teaching: Bridging the Lab-to-Field Gap

The most significant contribution of this research lies in demonstrating a viable solution to fill the “lab-to-field” gap in sports analysis in coaching and teaching. For decades, coaches and athletes have faced a trade-off: either conduct highly accurate analysis in an artificial, expensive laboratory environment or rely on subjective observation in the ecologically valid but data-poor training field. Capture4D offers a compelling bridge between these two extremes.

It is crucial to interpret the observed error margins within the context of practical coaching. While an average NMPJPE of 75.66 mm represents a positional discrepancy, this level of error is often smaller than the natural variability in an athlete’s movement between repetitions. More importantly, for a coach’s decision-making process, the preservation of kinematic patterns and temporal sequences is paramount. For instance, identifying whether an athlete’s shoulder rotation peak occurs before or after their hip rotation peak is a critical insight that is unaffected by minor absolute positional errors. The feedback for the coaches, particularly the high fidelity of the joint angle trajectories (Figure 3 and Figure 4), confirms that Capture4D reliably preserves these crucial temporal and relational patterns, making it a robust tool for practical, in-field technical evaluation.

Our findings show that the system provides data with sufficient accuracy to inform coaching decisions. For a coach, the precise absolute 3D position of an athlete’s elbow is often less critical than the pattern and timing of its movement. The high temporal agreement in joint angle trajectories (as seen in Figure 3 and Figure 4) confirms that Capture4D can reliably be used to assess an athlete’s kinetic chain sequencing.

Furthermore, the ability to perform a phase-based analysis and extract discrete, coach-centric metrics (Table 3) is a key advantage. A coach or teacher can use this system to answer critical performance questions directly on the court. For instance, by examining the ‘peak shoulder-hip separation angle’ at K1 and the subsequent ‘peak trunk rotation velocity’ leading into K2, a coach can quantitatively assess the efficiency of an athlete’s core in transferring power from the lower body to the upper limbs. A deficiency in this sequence could lead to specific corrective drills focusing on core stability and rotational power. Similarly, by monitoring the ‘peak shoulder internal rotation velocity’ during the K3 follow-through phase over multiple sessions, a coach could track potential indicators of fatigue or overload on the shoulder joint, which are critical for injury prevention strategies. This transforms quantitative analysis from a retrospective research activity into an immediate, actionable feedback loop that can be integrated into daily training routines. The comprehensive framework we developed for twelve distinct tennis movements, exemplified here by the serve, also underscores the system’s broad potential for holistic, long-term athlete monitoring.

### 4.2. Contextualizing the Contribution: Accessibility and Cost-Effectiveness

The primary contribution of this study, therefore, is not to challenge the sub-millimeter precision of OMC systems, but to validate a practical and accessible alternative that directly addresses these long-standing limitations. The goal of democratizing sports science by providing low-cost, easy-to-use tools for quantitative analysis is a significant and active area of research [30]. Our findings demonstrate that Capture4D, with its reliance on only a single standard camera and minimal setup time, represents a significant step toward this goal. By providing data of sufficient quality for kinematic pattern analysis and coaching feedback, it offers a solution that is orders of magnitude more cost-effective and operationally feasible for a vast majority of sports programs, from grassroots to professional levels.

### 4.3. Limitations and Future Directions

Despite the promising results, it is important to acknowledge the system’s limitations. The primary source of error is the inherent depth ambiguity of monocular vision. This is most evident when limbs move parallel to the camera’s image plane or during significant self-occlusion. While the Transformer architecture mitigates this by leveraging global context, high-speed movements can still introduce motion blur, challenging frame-by-frame estimation.

A primary limitation is the depth ambiguity inherent to monocular vision, particularly during phases of self-occlusion (e.g., the racket drop behind the back). While the Transformer model leverages learned priors to mitigate this, absolute errors in depth-dependent joint angles (like the elbow) can increase. However, the system successfully preserves the temporal kinematic chain, which is often the priority in coaching diagnostics.

Furthermore, regarding the validation metrics, this study primarily utilized NMPJPE, RMSE, and MAE, which are standard metrics in the field of computer vision for evaluating pose estimation accuracy. We acknowledge that from a biomechanical perspective, statistical agreement indicators such as correlation coefficients (e.g., Pearson’s r or ICC) are valuable for assessing waveform similarity. However, given the primary focus of this paper on validating the geometric reconstruction capability of the Transformer-based model, we did not include correlation analysis in the current scope. Future work will bridge this gap by incorporating comprehensive statistical agreement analyses to further strengthen the biomechanical validation.

Future research should focus on several key areas. First, further optimization of the Transformer model to specifically improve robustness to high-speed motion and severe occlusion would be beneficial. Second, exploring multi-sensor fusion, for instance, by integrating data from a single Inertial Measurement Unit (IMU) placed on the athlete, could dramatically improve accuracy with minimal impact on convenience. Finally, developing and validating sport-specific models, fine-tuned on extensive datasets of elite athletes in sports like tennis, golf, or baseball, could further enhance its performance and provide even more granular insights for coaches.

## 5. Conclusions

This study rigorously validated the Capture4D system as an effective and practical tool for the 3D kinematic analysis in tennis. The system provides accuracy within the acceptable range for applied biomechanical research and sport analysis while offering significant advantages in cost, portability, and ease of use over traditional OMC systems. By enabling coaches, teachers, and sports scientists to move quantitative analysis out of the laboratory and onto the court, Capture4D represents a significant step towards the democratization of sports motion analysis, empowering a data-informed approach to technique optimization and injury prevention.

## Figures and Tables

**Table 1:** Specifications of data capture systems.

Feature	Monocular System (Capture4D)	Optical Motion Capture (OMC)
System/Device	Single Standard RGB Camera	Qualisys High-speed Camera System
Sensor Type	CMOS RGB Sensor	Infrared High-speed Sensor
Resolution	1080p	High-precision 3D Spatial Coordinates
Sampling Rate	50 Hz	100 Hz
Markers	Markerless	Reflective Markers
Setup Process	Simple (Single camera, Zoom adjustable)	Complex (Calibration, Marker placement)
Environment	Flexible (Indoor/Outdoor, Natural Light)	Controlled Indoor Lab (10 m × 10 m)
Cost	Low (Consumer Grade)	High (Professional Grade)

**Table 2:** Comparison of overall 3D joint coordinate error (NMPJPE) between the Capture4D and OMC systems for each participant.

Participant ID	Avg. NMPJPE (mm)	Keyframe	Keyframe NMPJPE (mm)	L-Elbow Angle (OMC)	L-Elbow Angle (Capture4D)	R-Elbow Angle (OMC)	R-Elbow Angle (Capture4D)
1	76.51	219	59.34	144.32	115.65	83.23	96.58
2	70.85	199	76.13	140.36	131.95	40.48	80.15
3	72.98	252	82.05	150.29	133.19	65.51	104.15
4	70.94	160	64.73	122.46	118.38	56.44	83.86
5	78.93	58	49.67	154.87	148.34	148.84	153.85
6	79.71	175	59.78	98.55	104.42	62.41	90.14
7	69.53	153	92.74	145.67	132.66	67.89	98.22
8	72.53	188	86.29	152.30	137.42	55.47	94.43
9	88.27	180	85.67	159.21	139.26	68.04	95.75

**Table 3:** Definition of the key biomechanical phases of the tennis serve used for analysis.

Key Phase	Phase Name	Biomechanical Description
K0	Preparation	Athlete establishes a stable, balanced stance before initiating the motion.
K1	Backswing start	Backswing start marks the initiation of the tennis serve’s backswing phase. The athlete begins to move the racket from the stable.
K2	Peak of Backswing	The racket reaches its highest point; the shoulder achieves maximum external rotation to store elastic energy (i.e., the “Trophy Pose”).
K3	Contact Point	The moment of maximum racket velocity where the racket makes contact with the ball at the peak of the swing.
K4	Follow-through	The deceleration phase post-contact, where the arm continues across the body to safely dissipate force.
K5	Recovery	The final phase where the athlete returns to a balanced, ready position.

## Data Availability

Dataset available on request from the authors.
